# Comparative Analysis of the Effectiveness of Generic and Brand-Name Cefepime: A Multicenter Retrospective Cohort Study

**DOI:** 10.3390/life15020164

**Published:** 2025-01-24

**Authors:** Chih-Huang Li, Peng-Hao Chang, Ching-Tai Huang, Chip-Jin Ng

**Affiliations:** 1 Department of Emergency Medicine, Chang Gung Memorial Hospital Linkou Medical Center, Taoyuan 33342, Taiwan; chhli2002@gmail.com; 2 College of Medicine, Chang Gung University, Taoyuan 33302, Taiwan; sailinthesky@gmail.com; 3 Division of Infectious Diseases, Department of Internal Medicine, Chang Gung Medical Foundation Linkou Medical Center, Taoyuan 33342, Taiwan

**Keywords:** cefepime, generic drug, sepsis, effectiveness, safety, seizure

## Abstract

The clinical effectiveness of generic and brand-name cefepime in real-world settings remains unclear. Given the potential implications for healthcare costs and patient outcomes, this study aims to compare the efficacy and safety of these two formulations. We conducted a multicenter, retrospective cohort study (2017–2022), enrolling adults who received either generic or brand-name cefepime as initial monotherapy for at least three consecutive days. Demographics, comorbidities, disease severity, infection sites, cefepime dosage, and serial laboratory data were analyzed. Propensity score matching was employed to adjust key confounding variables. A total of 2370 patients were included, 877 receiving generic and 1493 receiving brand-name cefepime. After matching, no significant differences were observed in primary (in-hospital mortality, *p* = 0.841) or secondary outcomes (30-day mortality, readmission, length of stay, ICU stay, and incidence of seizures; all *p* > 0.05). Biomarker trends and clinical recovery patterns were comparable across groups. Receiving the maximal dosage or not did not alter outcomes among both groups. Generic cefepime showed comparable efficacy and safety to brand-name cefepime, offering a cost-effective alternative without compromising therapeutic outcomes.

## 1. Introduction

Cefepime, a fourth-generation cephalosporin antibiotic, exhibits broad-spectrum antibacterial activity against Gram-positive and Gram-negative bacteria [1,2]. It can penetrate various body tissues and exhibits enhanced stability against β-lactamases, rendering it a highly effective therapeutic option for a broad spectrum of infections, including pneumonia, urinary tract infections, intra-abdominal infections, and febrile neutropenia [3,4].

The effectiveness of cefepime against *Pseudomonas* spp. makes it a valuable therapeutic option in the hospital setting, where nosocomial infections and emerging bacterial resistance can be challenging to address [5,6]. This is especially relevant when other antibiotics are rendered ineffective because of bacterial resistance mechanisms, such as those related to extended-spectrum β-lactamases and AmpC enzymes [7,8,9]. Additionally, cefepime requires a less frequent dosing regimen and is relatively well-tolerated by patients, which contributes to cefepime often being preferred in clinical practice.

Various generic versions of cefepime were produced after the patent for the original expired. Bristol Myers Squibb (BMS) supplied brand-name cefepime (Maxipime) to the Taiwanese market before it was withdrawn in 2020. Supecef, a generic cefepime produced by China Chemical and Pharmaceutical Co., Ltd., Taipei, Taiwan was approved by the Taiwan Food and Drug Administration in 2007. Many medical facilities in Taiwan replaced Maxipime with Supecef after 2020. The Chang Gung Medical Foundation introduced Supecef as an alternative to Maxipime in 2017 and has been using Supecef as its only drug of choice since September, 2021. However, no study has yet compared the real-world efficacy of Supecef and Maxipime. Therefore, this multicenter, retrospective cohort study compared the therapeutic effectiveness and adverse effects of Supecef with those of Maxipime.

## 2. Materials and Methods

### 2.1. Study Design

This retrospective, propensity-score-matched study was conducted from January to December 2023. We collected patient data from 5 branches of the Chang Gung Medical Foundation. The study enrolled patients who were prescribed Supecef or Maxipime for 3 consecutive days after hospital admission. The primary outcome was in-hospital mortality. Secondary outcomes included 30-day mortality, intensive care unit (ICU) admission rate, length of ICU stay, and ICU mortality. We additionally assessed the trends of critical physical parameters and laboratory data, as well as the incidence of seizure—a serious complication of cefepime.

### 2.2. Data Collection

All data used in this study were retrieved from the Chang Gung Research Database, Taiwan’s largest multi-institutional electronic health record database, encompassing 7 Chang Gung Memorial Hospital institutions. From 2017 to 2022, we collected data from 5 branches of the Chang Gung Medical Foundation, namely, the Linkou, Keelung, Taipei, Chiayi, and Kaohsiung branches. Patients admitted to the emergency department or to wards were included in the study. Patients were included if (1) their age ≥ 18 years, (2) they had received either Maxipime or Supecef as initial monotherapy for at least 3 consecutive days, and (3) they had received a primary or secondary diagnosis of infectious disease upon admission that was based on the International Classification of Disease coding system. Patients were excluded if they had received both Supecef and Maxipime or had received other antibiotics within 7 days before receiving Supecef or Maxipime. This study was approved by the Institute Review Board of the Chang Gung Memorial Hospital (IRB no. 202200038B0).

### 2.3. Measurements

We retrieved the patients’ coma scale and vital sign data from the time of their emergency department or ward admission until discharge. We recorded the patients’ comorbidities, with these comorbidities including hypertension, hyperlipidemia, peripheral artery disease, heart failure, atrial fibrillation, chronic kidney disease, and malignancy, and we calculated Charlson Comorbidity Index scores by using the patients’ medical records. We retrieved laboratory data, including white blood cell (WBC) count; neutrophil and band percentage; platelet count; and levels of blood urea nitrogen (BUN), creatinine, sodium, potassium, total bilirubin, lactic acid, C-reactive protein (CRP), aspartate transferase (AST), and alanine transaminase (ALT). Estimated glomerular filtration rate (eGFR) and absolute neutrophil count (ANC) were calculated using established formulae. The site of infection was determined on the basis of the International Classification of Diseases code assigned at discharge. If patients were recorded as having both pneumonia and sepsis in the first 3 discharge diagnoses, both were listed in the results. The Sequential Organ Failure Assessment (SOFA) score was calculated using established criteria. Treatment duration and dosage information were retrieved from electronic medical records. The initiation of any anticonvulsant agent during the treatment period was considered to indicate an episode of new-onset seizure. We recorded the use of maximal recommended cefepime dosage regimens (e.g., 2 g every 8 h) or adjusted doses on the basis of eGFR to assess their impact on clinical outcomes and the occurrence of side effects.

### 2.4. Outcomes

The primary outcome was in-hospital mortality, which was defined on the basis of discharge status. The secondary outcomes included 30-day mortality, defined according to the national database on the causes of death, 30-day readmission rate, length of hospital stay, ICU admission rate, length of ICU stay, and ICU mortality (denominator: patients admitted to the ICU). We also compared the incidence of seizure and monitored serial parameters associated with infection status, including body temperature, leukocyte count, platelet count, and levels of creatinine, total bilirubin, CRP, AST, and ALT. All outcomes were compared between the 2 study groups.

### 2.5. Statistical Analysis

Regarding the patients’ baseline characteristics, categorical variables were compared using the chi-square test, and continuous variables were compared using independent sample t-tests; continuous variables that were not normally distributed (e.g., serum creatinine level and leukocyte count) were compared using the Mann–Whitney U-test. Possible confounding effects were minimized by using propensity score matching. All covariates listed in Table 1 were used to calculate propensity scores in the multivariable logistic regression model, including demographics (i.e., age and sex), initial vital signs, comorbidities, baseline laboratory data, infection focus, SOFA score at the index date, and whether a patient was admitted to the ICU on the index date. Each patient in the generic group was matched with a counterpart in the brand-name group. Missing data (Table 1) were imputed using the single expectation-maximization algorithm prior to propensity score matching and subsequent analyses.

The in-hospital outcomes in the propensity-score-matched cohort were compared between the generic and brand-name groups by using quantile regression for continuous variables and logistic regression for binary outcomes. The possible outcome dependency within the same matching pair was accounted for by using robust standard error, a method known as the generalized estimating equation approach. The analysis was further stratified according to whether the maximal antibiotic dose was received or not. We also retrieved data on the selected vital signs and laboratory results at different time points (i.e., baseline, 3 days, 7 days, 14 days, and 30 days). The changes in values from baseline to follow-up measurements were compared between the 2 groups by using a linear mixed-effects model, which included a random intercept, main effects of time and study group, and an interaction term between time and group. A significant interaction effect indicates a significant difference in the changes in values between 2 groups. A two-sided *p* value < 0.5 was considered to indicate significance, and statistical analyses were performed using SAS software, version 9.4 (SAS Institute Inc., Cary, NC, USA).

## 3. Results

**Figure 1:**
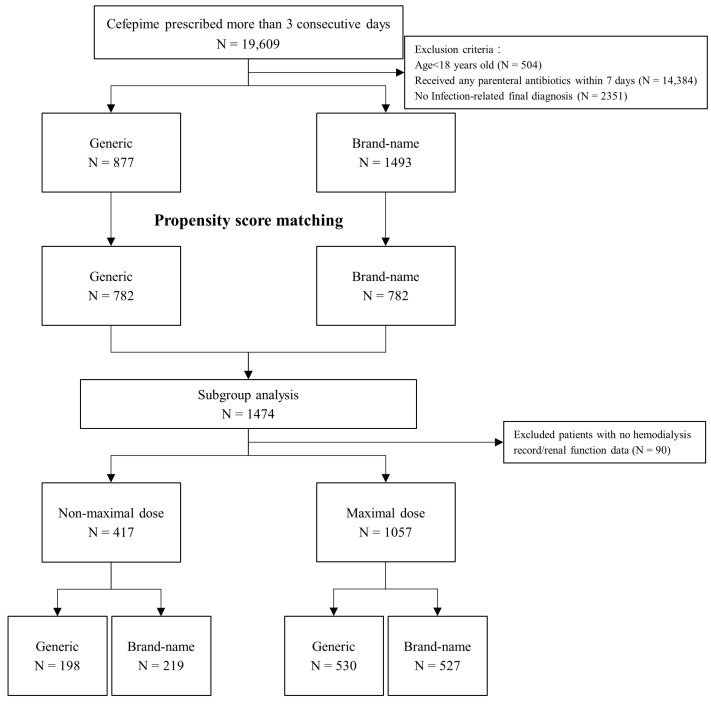
Flow chart for patient inclusion.

Figure 1 illustrates the patient selection process adopted in this retrospective study. A total of 19,609 patients were identified as having received cefepime (either generic or brand-name) for at least 3 consecutive days in the emergency department or a ward between 2017 and 2022. To isolate the effect of cefepime and avoid potential interactions with other medications, we excluded patients who received other antibiotics during the week prior to the initiation of cefepime. Moreover, patients younger than 18 years were excluded. The final sample comprised 2370 patients, of whom 877 received Supecef (generic cepefime) and 1493 received Maxipime (brand-name cefepime). Table 1 lists the patients’ baseline characteristics. The patients in the two groups had similar WBC counts, neutrophil and band percentages, ANC, and levels of BUN, potassium, total bilirubin, and lactic acid. However, they had significantly different levels of creatinine, sodium, AST, ALT, and CRP, as well as eGFRs. However, these parameters that significantly varied between the two groups are not of notable clinical importance. The two groups had similar SOFA scores. The patients in the generic group had a significantly lower incidence of respiratory tract infections (37.9% vs. 42.8%, *p* = 0.018) and a significantly higher incidence of urinary tract infections (18.7% vs. 14.7%, *p* = 0.01) than the patients in the brand-name group. The likelihood of receiving the maximal treatment dosage was comparable between the two groups (27.1% vs. 26.7%, *p* = 0.806). After propensity score matching was applied, each group contained 782 patients (Supplementary data Table S1). The two propensity-score-matched groups did not differ in terms of age, sex, initial vital signs, comorbidities, laboratory data, distribution of infection foci, SOFA scores, and treatment dosages.

Table 2 presents the outcome measurements for the two groups after propensity score matching. The primary outcome, in-hospital mortality, did not differ significantly between the two groups (17.5% vs. 17.1%, *p* = 0.841). Patients in the generic group exhibited a 30-day mortality of 18.5%, whereas those in the brand-name group exhibited a 30-day mortality of 17.5% (*p* = 0.599). Patients in the brand-name group stayed in hospital for a longer duration, but the difference was nonsignificant (median 12.0 days vs. 11.0 days, *p* = 0.132). ICU care was required by 39 patients in the generic group (5.2%) and by 48 in the brand-name group (6.5%), but the difference was nonsignificant (*p* = 0.318). The median ICU length of stay was 9.0 days in the generic group and 10.0 days in the brand-name group (*p* = 0.697). The number of ICU deaths was 25 in the generic group (32.9%) and 26 in the brand-name group (30.2%), but the difference was nonsignificant (*p* = 0.716). In addition, 14 patients in the generic group (1.8%) and 16 in the brand-name group (2.1%) developed seizures during their hospital stay, but the difference was nonsignificant (*p* = 0.713).

**Figure 2:**
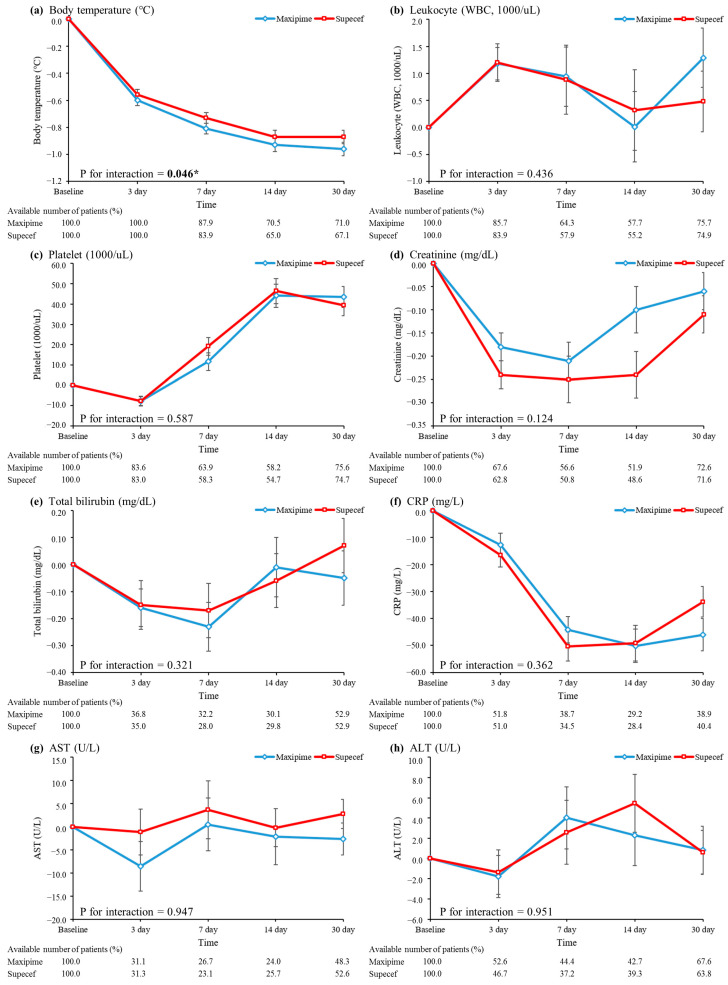
A comparison of the trends in inflammation and organ dysfunction biomarkers between patients using generic or brand-name cefepime. (**a**) Body temperature (**b**) leukocyte count, (**c**) platelet count (**d**) creatinine (**e**) total bilirubin (**f**) CRP (**g**) AST (**h**) ALT. * *p* < 0.05.

Figure 2 presents a comparison of the trends in inflammation and organ dysfunction biomarkers between the two groups for up to 30 days. The two groups exhibited similar temporal trends in body temperature, WBC and platelet counts, and levels of creatinine, total bilirubin, CRP, AST, and ALT (Figure 2a–h). The average body temperature decreased by up to 0.8 °C within 7 days. The average WBC count increased in the first 3 days and gradually decreased over the following 14 days in both groups. The platelet count increased by up to 50,000/µL within 14 days. The creatinine and CRP levels decreased by up to 0.3 mg/dL and 60 mg/L within 7 days, respectively. The total bilirubin, AST, and ALT levels did not change significantly over the treatment period.

**Figure 3:**
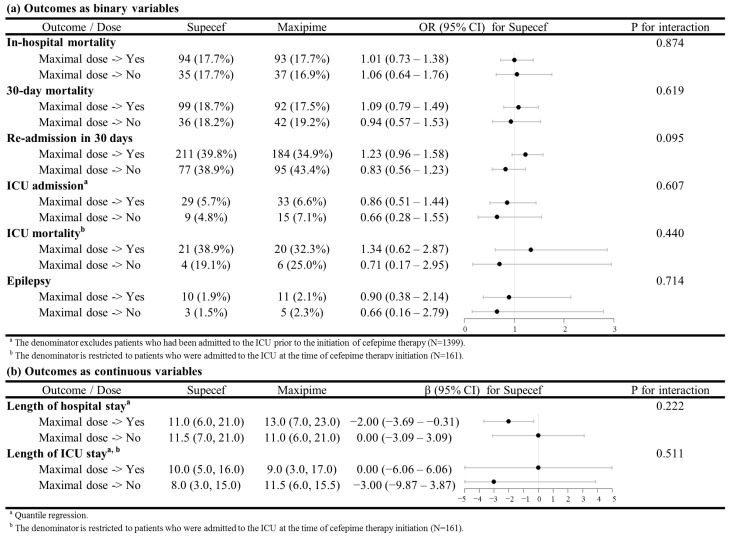
Comparison of patient outcomes between patients receiving the maximum dose and those receiving a lower dose. (**a**) Outcomes as binary variables, (**b**) outcomes as continuous variables. Comparison of patient outcomes between patients receiving the maximum dose and those receiving a lower dose.

We performed a subgroup analysis to investigate whether using the maximal recommended cefepime dosage regimen influenced patient outcomes in the two study groups. The results revealed that the administration of the maximal dose or a lower dose of Maxipime or Supecef resulted in similar in-hospital, 30-day, and ICU mortality rates; ICU admission rates; and lengths of hospital and ICU stay (Figure 3). The incidence of seizure was also similar between the patients receiving the maximal dose of Maxipime and Supecef.

## 4. Discussion

After the patent exclusivity of a brand-name antibiotic expires, a generic form of the antibiotic is often produced, which increases the cost-effectiveness of the antibiotic. However, the heterogeneity of evidence regarding the effectiveness of generic drugs has become an ongoing topic of debate among clinicians [10,11,12]. The present study compared the efficacy and safety profiles of the brand-name drug Maxipime and its generic counterpart Supecef, both of which are formulations of the antibiotic cefepime. This is the first comprehensive investigation to explore whether the generic formulation of a drug can be confidently utilized in place of the brand-name drug without compromising clinical outcomes. The clinical relevance of this study is enhanced by its emphasis on correcting the confounding effect of maximal dosage and evaluating crucial side effects, including seizure, in patients receiving cefepime.

Most of the baseline characteristics of the patients in the two groups were comparable, with no significant differences noted in key laboratory parameters such as WBC count, neutrophil and band percentage, ANC, and levels of BUN, potassium, total bilirubin, and lactic acid. The significant differences between the two groups in the baseline levels of creatinine, sodium, AST, ALT, and CRP as well as in eGFR were deemed to be clinically unimportant and did not translate into any notable differences in illness severity, as evidenced by the similar SOFA scores in the two groups. The treatment dose was similar between the groups, indicating that any observed differences in outcomes were not caused by disparities in dosage. Because brand-name cefepime was primarily used between 2017 and 2020, we started prescribing generic cefepime from 2020 until today. The patients’ characteristics did not change significantly throughout the study period. After confounding factors were adjusted for through propensity score matching, the baseline characteristics were balanced between the two cohorts (n = 782 each).

More than 60% of the patients in our study sample had sepsis. Respiratory tract infections accounted for nearly 40% of the infection focus; this incidence rate is comparable to that reported by a previous study on bloodstream infections and in [13]. However, we identified infections solely on the basis of the International Classification of Diseases coding system, which may not fully capture the comprehensive clinical criteria required for defining Sepsis-3 [14]. Nevertheless, we determined the mean SOFA score to be 5.2, which accurately reflects the severity of illness. In addition, the patients in our study exhibited a high comorbidity burden. Nearly 80% of the patients had an underlying malignancy, with a mean Charlson Comorbidity Index score of approximately 5.5. Additionally, nearly 30% of the patients presented with neutropenia. These findings suggest that the patients in this study had illnesses that were complicated and severe.

The in-hospital mortality rate—our primary outcome—did not differ significantly between the two groups, which confirms the therapeutic efficacy of generic cefepime. The severity of disease was reflected in the in-hospital mortality rate of 17% as well as the high SOFA scores of the patients. Notably, no significant differences were observed in the 30-day mortality rates between the two groups, indicating that the patients from both groups exhibited similar short-term survival outcomes. The two groups did not differ significantly in terms of secondary outcomes, that is, in overall length of stay in hospital and ICU, ICU admission rate, or ICU mortality. The generic group tended to have shorter hospital stays, but this difference was nonsignificant. Nevertheless, future studies with adequate statistical power should investigate whether treatment with the brand-name antibiotic is less efficient or associated with prolonged convalescence.

Cefepime-induced neurotoxicity manifests as a broad range of symptoms ranging from altered mental status to convulsions [15,16,17,18,19]. Our investigation focused on the development of seizure during hospitalization as a potential adverse event associated with cefepime treatment. No significant difference in the incidence of seizure was observed between the generic and brand-name groups. This finding has crucial implications for patient safety because neurological side effects can be extremely debilitating. Moreover, analysis of temporal trends in the levels of inflammation and organ dysfunction biomarkers indicated comparable responses between the two groups throughout the hospitalization and follow-up periods. Parameters such as body temperature, WBC and platelet count, and levels of creatinine, total bilirubin, CRP, AST, and ALT exhibited similar trends between the groups, highlighting the similarities in the therapeutic efficacies and patient recovery trajectories associated with generic and brand-name cefepime.

The optimal dosage of cefepime is typically determined by disease severity and renal function. The maximal dosage might be necessary to improve patient outcomes in specific clinical settings [7,20]. In our subgroup analysis, we focused on equivalence, avoiding comparing patients who did not receive the maximal dosage with those who did. This approach ensured that the outcomes in the two groups would not be confounded by any variations in dosage. Furthermore, a previous study identified higher plasma concentrations of cefepime as a potential risk factor for neurotoxicity, including seizure [21]. Reassuringly, our subgroup analysis revealed no increased incidence of seizure in the generic group, even in patients receiving the maximal dosage.

This study has some limitations inherent to its retrospective design. First, selection bias, which is a common concern in retrospective studies, could have compromised the generalizability of our findings. Although propensity score matching was employed to mitigate the effects of any potential bias, some residual confounding factors may remain unaccounted for. Second, excluding patients receiving antibiotics within one week might lessen the impact of antibiotic resistance. Third, we did not investigate the effect of individual microbiology and the drug resistance pattern. These factors should be addressed in future studies.

Nevertheless, this study demonstrates that Supecef is a viable alternative to Maxipime that yields comparable clinical outcomes without a significant increase in adverse events. These findings have considerable financial implications for health-care systems worldwide, indicating that broader adoption of generic medications can be employed to reduce health-care costs without compromising the quality of patient care. Overall, our research demonstrates the efficacy and safety of generic cefepime for the treatment of bacterial infections across diverse patient populations, including patients with complicated and severe illnesses. The findings indicate that generic cefepime formulations can be more broadly employed in hospital settings. Further studies should explore the potential reasons underlying the trend of longer treatment durations associated with Maxipime. Additionally, further studies should investigate whether the therapeutic equivalence observed between Supecef and Maxipime can be extended to other generic formulations of cefepime.

## 5. Conclusions

Cefepime remains a cornerstone medication for treating severe bacterial infections because of its broad-spectrum activity, favorable pharmacokinetics, and established clinical efficacy. The emergence of generic formulations of cefepime has further bolstered its status as an antibiotic in the hospital setting by providing a cost-effective therapeutic option without compromising quality. The discovery of an equivalence between brand-name and generic cefepime in this study can reassure health-care professionals and patients regarding the reliability of generic formulations, which is of particular importance in settings with limited health-care budgets. However, ongoing surveillance studies should continue to monitor the efficacy and safety of both generic and brand-name cefepime to ensure that treatment standards remain high and adaptations to resistance patterns are swift.

## Figures and Tables

**Table 1:** Selected baseline characteristics of patients using generic or brand-name cefepime. Missing data were imputed using the single expectation-maximization algorithm prior to propensity score matching and subsequent analyses.

Variable	Valid Number	Total	Generic	Brand-Name	*p*-Value
		(N = 2370)	(N = 877)	(N = 1493)	
Age (years)	2370	62.5 ± 14.7	63.9 ± 14.5	61.7 ± 14.7	<0.001
Male	2370	1268 (53.5%)	481 (54.9%)	787 (52.7%)	0.315
Initial vital signs					
Body temperature (°C)	2236	37.6 ± 0.9	37.6 ± 0.9	37.7 ± 0.9	0.012
Heart rate (beats/min)	2268	96.3 ± 16.4	95.0 ± 16.1	97.0 ± 16.6	0.006
Respiratory rate (breaths/min)	2269	18.6 ± 2.8	18.3 ± 2.7	18.8 ± 2.9	<0.001
Systolic blood pressure (mmHg)	2296	120.3 ± 19.2	120.6 ± 19.3	120.2 ± 19.2	0.635
Diastolic blood pressure (mmHg)	2296	71.0 ± 11.3	70.9 ± 11.3	71.1 ± 11.3	0.763
Mean arterial pressure (mmHg)	2296	87.4 ± 12.8	87.5 ± 13.0	87.4 ± 12.7	0.952
Comorbidity (n, %)					
Hypertension	2370	1034 (43.6%)	420 (47.9%)	614 (41.1%)	0.001
Hyperlipidemia	2370	492 (20.8%)	205 (23.4%)	287 (19.2%)	0.016
Peripheral artery disease	2370	57 (2.4%)	18 (2.1%)	39 (2.6%)	0.391
Heart Failure	2370	200 (8.4%)	78 (8.9%)	122 (8.2%)	0.541
Atrial fibrillation	2370	135 (5.7%)	53 (6.0%)	82 (5.5%)	0.576
Chronic kidney disease	2370	374 (15.8%)	152 (17.3%)	222 (14.9%)	0.112
Malignancy	2370	1879 (79.3%)	697 (79.5%)	1182 (79.2%)	0.859
CCI score	2370	5.2 ± 3.1	5.4 ± 3.1	5.1 ± 3.1	0.012
Index Dialysis	2370	79 (3.3%)	24 (2.7%)	55 (3.7%)	0.215
Laboratory					
Leukocyte (WBC, count, 1000/uL)	2311	2.8 (0.9, 10.2)	2.7 (1.0, 10.6)	2.9 (0.9, 10.1)	0.778
Neutrophil (%)	2111	56.1 ± 30.4	56.4 ± 30.2	55.9 ± 30.5	0.711
Band (%)	676	2.0 (1.0, 4.0)	2.0 (1.0, 4.0)	2.0 (1.0, 4.3)	0.411
Platelet (1000/uL)	2306	152.4 ± 127.1	157.2 ± 128.4	149.5 ± 126.3	0.150
BUN (mg/dL)	1831	26.1 ± 21.4	27.2 ± 22.4	25.5 ± 20.7	0.099
Creatinine (mg/dL)	2210	0.9 (0.6, 1.3)	0.9 (0.7, 1.3)	0.9 (0.6, 1.3)	0.044
Sodium (Na, mEq/L)	2176	134.3 ± 5.7	133.8 ± 5.8	134.6 ± 5.6	0.002
Potassium (K, mEq/L)	2188	3.8 ± 0.6	3.8 ± 0.6	3.8 ± 0.6	0.112
Total bilirubin (mg/dL)	1332	0.7 (0.5, 1.1)	0.7 (0.5, 1.1)	0.7 (0.5, 1.1)	0.770
Lactic acid (mg/dL)	816	20.1 ± 14.6	20.9 ± 15.4	19.6 ± 14.0	0.205
CRP (mg/L)	1789	101.5 ± 85.8	107.7 ± 90.4	97.5 ± 82.5	0.017
AST (U/L)	881	30.0 (21.0, 47.0)	29.0 (19.0, 45.0)	31.0 (22.0, 50.0)	0.010
ALT (U/L)	1892	24.0 (15.0, 42.0)	22.0 (14.0, 38.0)	25.0 (16.0, 44.0)	0.005
eGFR (mL/min/1.73 m^2^)	2210	83.1 ± 48.1	80.2 ± 46.3	84.8 ± 49.0	0.030
Low absolute neutrophil count	2110	627 (29.7%)	237 (29.2%)	390 (30.1%)	0.670
Infection focus					
Respiratory Tract Infection (RTI)	2370	971 (41.0%)	332 (37.9%)	639 (42.8%)	0.018
Urinary Tract Infection (UTI)	2370	383 (16.2%)	164 (18.7%)	219 (14.7%)	0.010
Intra-Abdominal Infection (IAI)	2370	212 (9.0%)	80 (9.1%)	132 (8.8%)	0.817
Skin and Soft Tissue Infection (SSTI)	2370	99 (4.2%)	35 (4.0%)	64 (4.3%)	0.728
Sepsis	2370	1564 (66.0%)	597 (68.1%)	967 (64.8%)	0.101
Other	2370	388 (16.4%)	117 (13.3%)	271 (18.2%)	0.002
SOFA score	2370	5.2 ± 2.1	5.2 ± 2.1	5.2 ± 2.1	0.611
Non-maximal dose	2229	601 (27.0%)	384 (27.1%)	217 (26.7%)	0.806
Maximal dose		1628 (73.0%)	1031 (72.9%)	597 (73.3%)	

**Table 2:** Patient outcomes of patients using generic or brand-name cefepime.

Outcome	Generic	Brand-Name	β or OR (95% CI)	*p*-Value
	(N = 782)	(N = 782)		
In-hospital mortality	137 (17.5%)	134 (17.1%)	1.03 (0.79–1.34)	0.841
30-day mortality	145 (18.5%)	137 (17.5%)	1.07 (0.83–1.39)	0.599
Length of hospital stay	11.0 (6.0, 21.0)	12.0 (7.0, 21.0)	−1.00 (−2.30–0.30)	0.132
ICU admission percentage	39 (5.2%)	48 (6.5%)	0.80 (0.52–1.24)	0.318
Length of ICU stay	9.0 (4.5, 16.0)	10.0 (4.0, 17.0)	−1.00 (−6.06–4.06)	0.697
ICU mortality	25 (32.9%)	26 (30.2%)	1.13 (0.58–2.20)	0.716
30-day re-hospitalization	312 (39.9%)	297 (38.0%)	1.08 (0.89–1.33)	0.437
Seizure	14 (1.8%)	16 (2.1%)	0.87 (0.42–1.80)	0.713

## Data Availability

The datasets used and analyzed during the current study are available from the corresponding author on reasonable request.

## Supplementary Materials

The following supporting information can be downloaded at: https://www.mdpi.com/article/10.3390/life15020164/s1, Table S1. Selected baseline characteristics of the patients with use of the generic or brand-name cefepime after propensity score matching.
