# Supplementary material for: Comparative Analysis of the Effectiveness of Generic and Brand-Name Cefepime: A Multicenter Retrospective Cohort Study
Source: Life (Basel). 2025 Jan 24;15(2):164. doi: 10.3390/life15020164 (PMC11856423; doi:10.3390/life15020164)
Supplement: Supplementary file 1 [file life-15-00164-s001.zip › life-3401708-supplementary.pdf]

Supplementary data Table S1. Selected baseline characteristics of the patients with use of the generic or brand-name cefepime after propensity score matching

Variable	Supecef (N = 782)	Maxipime (N = 782)	*P*-value
Age (years)	64.1 ± 14.3	63.9 ± 13.7	0.831
Male	433 (55.4%)	430 (55.0%)	0.879
Initial vital signs			
Body temperature (°C)	37.6 ± 0.9	37.6 ± 1.0	0.272
Heart rate (beats/min)	95.4 ± 16.1	95.7 ± 16.6	0.701
Respiratory rate (breaths/min)	18.3 ± 2.8	18.4 ± 2.5	0.286
Systolic blood pressure (mmHg)	120.5 ± 19.6	120.5 ± 19.1	0.995
Diastolic blood pressure (mmHg)	70.7 ± 11.5	71.0 ± 11.3	0.692
Mean arterial pressure (mmHg)	87.3 ± 13.2	87.5 ± 12.8	0.815
Comorbidity (n, %)			
Hypertension	372 (47.6%)	372 (47.6%)	1.000
Hyperlipidemia	177 (22.6%)	185 (23.7%)	0.632
Peripheral artery disease	16 (2.1%)	18 (2.3%)	0.729
Heart Failure	67 (8.6%)	69 (8.8%)	0.858
Atrial fibrillation	48 (6.1%)	47 (6.0%)	0.916
Chronic kidney disease	129 (16.5%)	138 (17.7%)	0.545
Malignancy	621 (79.4%)	616 (78.8%)	0.756
CCI score	5.5 ± 3.1	5.6 ± 3.1	0.768
Index Dialysis	22 (2.8%)	23 (2.9%)	0.880
Laboratory			
Leukocyte (WBC, count, 1000/uL)	2.9 (1.0, 10.8)	3.3 (1.1, 11.0)	0.714
Neutrophil (%)	56.0 ± 30.3	56.7 ± 29.9	0.659
Band (%)	2.9 (1.0, 5.3)	3.0 (1.0, 5.4)	0.864
Platelet (1000/uL)	160.2 ± 128.5	161.1 ± 131.1	0.884
BUN (mg/dL)	25.9 ± 21.3	25.9 ± 21.8	0.956
Creatinine (mg/dL)	0.9 (0.7, 1.3)	0.9 (0.7, 1.4)	0.711
Sodium (Na, mEq/L)	133.9 ± 5.7	134.0 ± 5.6	0.837

Potassium (K, mEq/L)	3.8 ± 0.6	3.8 ± 0.6	0.668
Total bilirubin (mg/dL)	0.8 (0.4, 1.5)	0.8 (0.4, 1.4)	0.989
Lactic acid (mg/dL)	18.0 ± 14.9	18.5 ± 14.8	0.517
CRP (mg/L)	106.0 ± 87.9	104.7 ± 84.6	0.773
AST (U/L)	29.0 (14.0, 58.0)	34.0 (15.0, 65.0)	0.059
ALT (U/L)	25.0 (14.0, 46.0)	26.0 (15.0, 45.4)	0.674
eGFR (mL/min/1.73m^2)	79.3 ± 44.9	79.4 ± 46.1	0.984
Low ANC	230 (29.4%)	234 (29.9%)	0.825
Infection focus			
Respiratory Tract Infection (RTI)	307 (39.3%)	295 (37.7%)	0.533
Urinary Tract Infection (UTI)	146 (18.7%)	146 (18.7%)	1.000
Intra-Abdominal Infection (IAI)	71 (9.1%)	74 (9.5%)	0.794
Skin and Soft Tissue Infection (SSTI)	33 (4.2%)	34 (4.4%)	0.901
Sepsis	526 (67.3%)	516 (66.0%)	0.592
Other	104 (13.3%)	97 (12.4%)	0.597
SOFA score	5.2 ± 2.2	5.2 ± 2.1	1.000
Non-Maximal dose	219 (29.4%)	198 (27.2%)	0.358
Maximal dose	527 (70.6%)	530 (72.8%)	
